# Rateless Codes-Based Secure Communication Employing Transmit Antenna Selection and Harvest-To-Jam under Joint Effect of Interference and Hardware Impairments

**DOI:** 10.3390/e21070700

**Published:** 2019-07-16

**Authors:** Phu Tran Tin, Tan N. Nguyen, Nguyen Q. Sang, Tran Trung Duy, Phuong T. Tran, Miroslav Voznak

**Affiliations:** 1 VSB—Technical University of Ostrava, 17. listopadu 15/2172, 708 33 Ostrava, Czech Republic; 2 Faculty of Electronics Technology, Industrial University of Ho Chi Minh City, Ho Chi Minh City 700000, Vietnam; 3 Department of Electrical and Electronic Engineering, Duy Tan University, DaNang City 550000, Vietnam; 4 Department of Telecommunications, Posts and Telecommunications Institute of Technology, Ho Chi Minh City 700000, Vietnam; 5 Wireless Communications Research Group, Faculty of Electrical and Electronics Engineering, Ton Duc Thang University, Ho Chi Minh City 700000, Vietnam

**Keywords:** rateless codes, transmit antenna selection, energy harvesting, co-channel interference, hardware impairments

## Abstract

In this paper, we propose a rateless codes-based communication protocol to provide security for wireless systems. In the proposed protocol, a source uses the transmit antenna selection (TAS) technique to transmit Fountain-encoded packets to a destination in presence of an eavesdropper. Moreover, a cooperative jammer node harvests energy from radio frequency (RF) signals of the source and the interference sources to generate jamming noises on the eavesdropper. The data transmission terminates as soon as the destination can receive a sufficient number of the encoded packets for decoding the original data of the source. To obtain secure communication, the destination must receive sufficient encoded packets before the eavesdropper. The combination of the TAS and harvest-to-jam techniques obtains the security and efficient energy via reducing the number of the data transmission, increasing the quality of the data channel, decreasing the quality of the eavesdropping channel, and supporting the energy for the jammer. The main contribution of this paper is to derive exact closed-form expressions of outage probability (OP), probability of successful and secure communication (SS), intercept probability (IP) and average number of time slots used by the source over Rayleigh fading channel under the joint impact of co-channel interference and hardware impairments. Then, Monte Carlo simulations are presented to verify the theoretical results.

## 1. Introduction

Physical-layer security (PLS) [1,2,3,4] has attracted much attention of the researchers as an efficient method to attain security. Due to the simple implementation, i.e., only exploiting characteristics of wireless medium such as link distance and channel state information (CSI), PLS can be implemented efficiently in wireless sensor networks (WSNs), internet-of-things (IoT) networks, etc. [5,6,7,8]. To enhance the secrecy performance, diversity transmission methods can be employed. In [9,10,11,12], MIMO-based transmit–receive methods such as Transmit Antenna Selection-Maximal Ratio Combining (TAS-MRC), Maximal Ratio Transmission-MRC (MRT-MRC), MRT-Selection Combining (MRT-SC), MRT-SC were proposed and analyzed. In addition, performance of secure communication protocols can be also enhanced with cooperative relaying methods [13,14,15,16]. In [17,18,19,20], the authors proposed cooperative jamming (CJ) techniques to reduce quality of the eavesdropping channels, where friendly jammers are employed to generate artificial noises on the eavesdropper, and the legitimate receivers have to cooperate with the jammers to remove the interference in the received signals. The results presented that the schemes which combine the diversity transmission and the jamming techniques outperform the conventional cooperative ones without using CJ. However, energy efficiency may become a critical issue when the jammer nodes continuously transmit the artificial noises by using their own energy. Recently, radio frequency (RF) energy harvesting (EH) is an efficient method to prolong lifetime for wireless networks [21,22,23,24]. Particularly, the wireless devices can harvest energy from full-energy nodes [21,22] or from power stations deployed in networks [25,26] or even from co-channel interferences caused by outside sources [27,28]. References [20,29] proposed and analyzed performance of RF-EH-based secure communication protocols. To support energy for the jammer nodes, the authors of [20,29] proposed harvest-to-jam (HJ) methods, where the cooperative jammers harvest energy from the RF signals, and then use it to generate artificial noises.

Rateless codes or Fountain codes (FCs) [30,31,32,33] have drawn much attention due to their simple implementation. In FCs, a transmitter uses Fountain encoder to generate a limitless number of encoded packets, and then transmit them to intended receivers. If the receivers can receive a sufficient number of the encoded packets, they can recover the original message of the transmitter. Due to broadcast of the wireless channels, the encoded packets can be overheard by eavesdroppers. Therefore, the security becomes a critical issue for the FCs-based communication systems. Recently, some published works considering the secure communication protocols with FCs have been reported in [34,35,36]. In [34], the authors proposed a secure delivery scheme, in which the security can be achieved if the legitimate user receives enough Fountain packets before the eavesdropper. In [35], a dynamic Fountain-encoded at a transmitter was proposed to enhance the data security. The authors of [36] proposed a FC-based cooperative relay protocol. In [36], the source and the jammer cooperate to remove the interference components in the received signals at the destination. Reference [37] proposed an efficient FCs-based multicast scenario to achieve security for Internet of Things (IoT) systems.

In this paper, we propose a FCs based secure communication protocol, where a multi-antenna source selects its best antenna to transmit the encoded packets to a single-antenna destination, in presence of a single-antenna eavesdropper who attempts to overhear the source information. When the destination can receive sufficient encoded packets for decoding the original data, it would send a feedback to the source to terminate the transmission. As a result, to obtain the secure transmission, the destination must receive a sufficient number of the encoded packets before the eavesdropper. Otherwise, the original information is intercepted. The main contributions of this paper can be summarized as follows:

- To the best of our knowledge, we first propose the FCs based communication protocol using the harvest-to-jam based cooperative jamming technique to reduce the quality of the eavesdropping link. Different with [34,35,36,37], we propose a cooperative jamming technique, where a cooperative jammer node harvests energy from the RF signals of the source and the interference sources to generate noises to the eavesdropper. Different with our previous works [38,39], in the proposed protocol, there exist interference sources in the network that cause co-channel interferences on both the destination and the eavesdropper.
- Until now, almost published works related to secrecy performance evaluation have assumed that the transceiver hardware of the wireless devices is perfect. However, in practice, it is not perfect due to phase noise, I/Q imbalance (IQI), amplifier non-linearity [40,41,42,43]. In this paper, the joint impact of hardware noises and co-channel interference on the system performances is investigated.
- For performance evaluation, we derive exact closed-form expressions of outage probability (OP), probability of successful and secure communication (SS), intercept probability (IP) and average number of the time slots used by the source over Rayleigh fading channel. The closed-form formulas are easy-to-compute, and hence they can be easily used to design and optimize the considered system. In addition, all of the derived expressions are verified by Monte Carlo simulations.

The rest of this paper is organized as follows. The system model of the proposed protocols is described in Section 2. In Section 3, we evaluate performance of the proposed scheme. The simulation results are shown in Section 4. Finally, this paper is concluded in Section 5.

## 2. System Model

**Figure 1:**
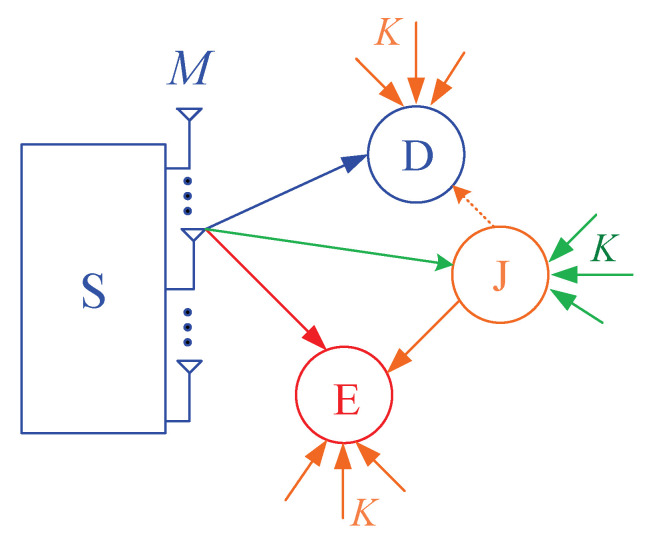
System model of the proposed scheme.

Figure 1 illustrates the system model of the proposed protocol, where the source node (S) equipped with *M* antennas communicates with the single-antenna destination (D), in presence of the single-antenna eavesdropper (E) who attempts to overhear the source data. All of the receivers such as D and E are suffered from co-channel interference caused by *K* ambient sources (denoted by $I_{1},I_{2},\ldots ,I_{K}$). To reduce the quality of the eavesdropping link, the cooperative jamming technique can be used, where the single antenna jammer (J) is employed to continuously generate the artificial noises to E. We assume that the nodes D and J are close with each other so that D can remove the co-channel interference generated by J [38]. Moreover, the jammer (J) uses the energy harvested from the RF signals of the source and the interference sources for transmitting the jamming signals.

The source divides its original data into *L* packets which are encoded appropriately to create the encoded packets. Then, at each time slot, the source uses the TAS technique to send each encoded packet to the destination. At the same time, the eavesdropper tries to receive the encoded packet. The destination and the eavesdropper are assumed to be able to successfully obtain the original data if they can correctly receive at least *H* encoded packets, where $H=\left(1+\varepsilon \right)L$, and ε is the decoding overhead which depends on concrete code design. Moreover, after the destination receives sufficient number of the encoded packets, it will send an ACK message to inform the source to stop the data transmission. In this case, if the eavesdropper cannot obtain enough number of the encoded packets, it cannot obtain the source data. Otherwise, the original data of the source will be intercepted.

Let us consider the data transmission at an arbitrary time slot. Let $h_{S_{m}D}$, $h_{S_{m}E}$ and $h_{S_{m}J}$ denote channel coefficients between the mth antenna of the source and the nodes D, E and J, respectively, where m=1,2,…,M. We also denote $h_{I_{k}D},$ $h_{I_{k}E},$ $h_{I_{k}J}$ and $h_{\mathrm{JE}}$ as channel gains of the $I_{k}\to D,$ $I_{k}\to E,$ $I_{k}\to J$ and J→E links, respectively, where k=1,2,…,K. We assume that all of the link channels are block and flat Rayleigh fading which keeps constant in a time slot but independently changes over time slots. Therefore, the channel gains $\gamma_{\mathrm{XY}}=|h_{\mathrm{XY}}{|}^{2},\left(X,Y\in \left\{S_{m},D,E,J,I_{k}\right\}\right)$ are exponential random variables (RVs) whose cumulative distribution function (CDF) and probability density function (PDF) are given, respectively as

(1) $$\begin{array}{c} F_{\gamma_{\mathrm{XY}}}\left(z\right)=1-\exp\left(-\lambda_{\mathrm{XY}}x\right),\\ f_{\gamma_{\mathrm{XY}}}\left(z\right)=\lambda_{\mathrm{XY}}\exp\left(-\lambda_{\mathrm{XY}}x\right), \end{array}$$

where $\lambda_{\mathrm{XY}}$ is a parameter of $\gamma_{\mathrm{XY}},$ i.e., $\lambda_{\mathrm{XY}}=1/E\left\{\gamma_{\mathrm{XY}}\right\},$ and $E\left\{.\right\}$ is an expected operator. We can assume that the RVs $\gamma_{S_{m}D}\left(\gamma_{S_{m}E},\gamma_{S_{m}J}\right)$ are independent and identical, i.e., $\lambda_{S_{m}D}=\lambda_{\mathrm{SD}}$ $\left(\lambda_{S_{m}E}=\lambda_{\mathrm{SE}},\lambda_{S_{m}J}=\lambda_{\mathrm{SJ}}\right)$ for all *m*. On the contrary, the RVs $\gamma_{I_{k}D}\left(\gamma_{I_{k}E},\gamma_{I_{k}J}\right)$ are assumed to be independent and non-identical, i.e., $\lambda_{I_{k}D}\neq \lambda_{I_{l}D}$ $\left(\lambda_{I_{k}E}\neq \lambda_{I_{l}E},\lambda_{I_{k}J}\neq \lambda_{I_{l}J}\right)$ as k≠l, where $l\in \left\{1,2,\ldots ,K\right\}$.

With the TAS technique, the source selects the best transmit antenna to send the encoded packet to the destination, using the following method:

(2) $$\begin{array}{c} b=\underset{m=1,2,\ldots ,M}{\arg\max}\left(\gamma_{S_{m}D}\right), \end{array}$$

where $b\in \left\{1,2,\ldots ,M\right\}$.

Moreover, the CDF of $\gamma_{S_{b}D}$ can be obtained as

(3) $$\begin{array}{cc} F_{\gamma_{S_{b}D}}\left(x\right) & =\mathrm{Pr}\left(\max_{m=1,2,\ldots ,M}\left(\gamma_{S_{m}D}\right)<x\right)\\ & ={\left[1-\exp\left(-\lambda_{\mathrm{SD}}x\right)\right]}^{M}\\ & =1+\sum_{m=1}^{M}{\left(-1\right)}^{m}C_{M}^{m}\exp\left(-m\lambda_{\mathrm{SD}}x\right), \end{array}$$

where $C_{M}^{m}=M!/m!/\left(M-m\right)!$ is a binomial coefficient.

Let us denote T as a block time of each time slot: a duration of $\alpha T\left(0\leq \alpha \leq 1\right)$ is used for the jammer node to harvest the energy from the source and the interference sources, and the remaining time $\left(\left(1-\alpha \right)T\right)$ is spent for the data transmission. Then, the energy harvested by the jammer is expressed as

(4) $$\begin{array}{c} \mathrm{EH}=\eta \alpha T\left(P_{S}\gamma_{S_{b}J}+\sum_{k=1}^{K}P_{I_{k}}\gamma_{I_{k}J}\right), \end{array}$$

where $\eta \left(0\leq \eta \leq 1\right)$ is an energy conversion efficiency, $P_{S}$ and $P_{I_{k}}$ are transmit power of the source (S) and the interference sources $I_{k}$, respectively.

Next, the average transmit power of the jammer used for the data transmission phase can be formulated by

(5) $$\begin{array}{c} P_{J}=\frac{\mathrm{EH}}{\left(1-\alpha \right)T}=\chi \left(P_{S}\gamma_{S_{b}J}+\sum_{k=1}^{K}P_{I_{k}}\gamma_{I_{k}J}\right), \end{array}$$

where $\chi =\eta \alpha /\left(1-\alpha \right)$.

It is worth noting that the implementation of the TAS method is simpler than that of the MRT method because it only requires the index of the best antenna which can be feed-backed by the destination (not feedback all of the channel state information (CSI) as in MRT). Moreover, the best transmit antenna selection can be performed before the EH phase, and the time used for this process can be ignored as compared with the EH and packet transmission phases. Finally, the source uses the selected antenna during each time slot for both the EH and data transmission purposes due to scheduling issues, e.g., the source uses the remaining antennas to serve other destinations.

Let us denote *U* as the length of each encoded packet. If the source sends the signal $x_{S}\left[l\right]\left(l=1,2,\ldots ,U\right)$ to the destination, the received signals at the destination and the eavesdropper can be expressed, respectively as

(6) $$\begin{array}{c} y_{D}=\sqrt{P_{S}}h_{S_{b}D}\left(x_{S}\left[l\right]+\nu_{D}\left[l\right]\right)+\sqrt{P_{J}}h_{\mathrm{JD}}x_{J}\left[l\right]+\sum_{k=1}^{K}\sqrt{P_{I_{k}}}h_{I_{k}D}x_{I_{k}}\left[l\right]+n_{D}\left[l\right],\\ y_{E}=\sqrt{P_{S}}h_{S_{b}E}\left(x_{S}\left[l\right]+\nu_{E}\left[l\right]\right)+\sqrt{P_{J}}h_{\mathrm{JE}}x_{J}\left[l\right]+\sum_{k=1}^{K}\sqrt{P_{I_{k}}}h_{I_{k}E}x_{I_{k}}\left[l\right]+n_{E}\left[l\right], \end{array}$$

where l=1,2,…,U, $\nu_{D}\left[l\right]$ and $\nu_{E}\left[l\right]$ are hardware noises caused by impairments, $x_{J}\left[l\right]$ and $x_{I_{k}}\left[l\right]$ are signals transmitted by the nodes J and $I_{k}$, respectively, and $n_{D}\left[l\right]$ and $n_{E}\left[l\right]$ are additive white Gaussian noises (AWGNs) at D and E, respectively. The hardware noises $\nu_{D}\left[l\right]$ and $\nu_{E}\left[l\right]$ can be modeled as Gaussian RVs with zero-mean and variances of $\kappa_{D}^{2}$ and $\kappa_{E}^{2}$, respectively, where $\kappa_{D}^{2}$ and $\kappa_{E}^{2}$ is total hardware impairment levels of the $S_{b}\to D$ and $S_{b}\to E$ links, respectively.

Because the nodes D and J are close with each other so that we can assume that D knows $x_{J}\left[l\right]$, $h_{\mathrm{JD}}$ and $P_{J}$ via securely exchanging local messages with J. Therefore, D can remove the interference component $\sqrt{P_{J}}h_{\mathrm{JD}}x_{J}\left[l\right]$ from the received signal $y_{D}$. Once D can perfectly remove the interference, the instantaneous signal-to-interference-plus-noise ratio (SINR) received by the destination under joint impact of co-channel interference and hardware impairments can be formulated as [44]

(7) $$\begin{array}{cc} \Psi_{D} & =\frac{P_{S}\gamma_{S_{b}D}}{\kappa_{D}^{2}P_{S}\gamma_{S_{b}D}+\sum_{k=1}^{K}P_{I_{k}}\gamma_{I_{k}D}+N_{0}}\\ & =\frac{Q_{S}\gamma_{S_{b}D}}{\kappa_{D}^{2}Q_{S}\gamma_{S_{b}D}+\sum_{k=1}^{K}Q_{I_{k}}\gamma_{I_{k}D}+1}, \end{array}$$

where $N_{0}$ is variance of additive noises $n_{D}\left[l\right]$ which are assumed to be same at all of the receivers, $Q_{S}=P_{S}/N_{0}$ and $Q_{I_{k}}=P_{I_{k}}/N_{0}$.

Because the eavesdropper cannot remove the jamming signals, the instantaneous SINR obtained at this node is given as

(8) $$\begin{array}{c} \Psi_{E}=\frac{P_{S}\gamma_{S_{b}E}}{\kappa_{E}^{2}P_{S}\gamma_{S_{b}E}+P_{J}\gamma_{\mathrm{JE}}+\sum_{k=1}^{K}P_{I_{k}}\gamma_{I_{k}D}+N_{0}}. \end{array}$$

Substituting (5) into (8), we obtain

(9) $$\begin{array}{c} \Psi_{E}=\frac{Q_{S}\gamma_{S_{b}E}}{\kappa_{E}^{2}Q_{S}\gamma_{S_{b}E}+\chi \left(\;Q_{S}\gamma_{S_{b}J}+\sum_{k=1}^{K}Q_{I_{k}}\gamma_{I_{k}J}\right)\gamma_{\mathrm{JE}}+\sum_{k=1}^{K}Q_{I_{k}}\gamma_{I_{k}E}+1}. \end{array}$$

Next, we can give expressions of the data rate for the data and eavesdropping links, respectively by

(10) $$\begin{array}{c} C_{D}=\left(1-\alpha \right){\mathrm{Tlog}}_{2}\left(1+\Psi_{D}\right),\\ C_{E}=\left(1-\alpha \right){\mathrm{Tlog}}_{2}\left(1+\Psi_{E}\right). \end{array}$$

Assume that each encoded packet can be decoded successfully if the achievable data rate is higher than a predetermined target rate (denoted by $C_{\mathrm{th}}$). Otherwise, the encoded packet cannot be received correctly. Hence, the probability that the destination cannot receive one encoded packet correctly is formulated as

(11) $$\begin{array}{c} \mathrm{Pr}\left(C_{D}<C_{\mathrm{th}}\right)=\mathrm{Pr}\left(\Psi_{D}<\theta_{\mathrm{th}}\right)\overset{\Delta }{\;=}\rho_{D}, \end{array}$$

where

(12) $$\begin{array}{c} \theta_{\mathrm{th}}=2^{\frac{C_{\mathrm{th}}}{\left(1-\alpha \right)T}}-1. \end{array}$$

Note that the probability of the successful decoding for one encoded packet at D is $\mathrm{Pr}\left(C_{D}\geq C_{\mathrm{th}}\right)=1-\rho_{D}$. Similarly, the probability that one encoded packet can be received correctly and incorrectly by the eavesdropper is given, respectively as

(13) $$\begin{array}{c} \mathrm{Pr}\left(C_{E}<C_{\mathrm{th}}\right)=\mathrm{Pr}\left(\Psi_{E}<\theta_{\mathrm{th}}\right)\overset{\Delta }{\;=}\rho_{E},\\ \mathrm{Pr}\left(C_{E}\geq C_{\mathrm{th}}\right)=\mathrm{Pr}\left(\Psi_{E}\geq \theta_{\mathrm{th}}\right)=1-\rho_{E}. \end{array}$$

Considering a delay-constrained system where the maximum number of time slots that can be used for transmitting the encoded packets is limited by $N_{\mathrm{th}}\left(N_{\mathrm{th}}\geq H\right)$. This means that the destination cannot recover the original data if it cannot successfully receive *H* encoded packets within $N_{\mathrm{th}}$ time slots. Let us denote $N_{S}\left(H\leq N_{S}\leq N_{\mathrm{th}}\right)$ as the number of time slots used by the source (or the number of the encoded packets sent by the source), $N_{D}$ and $N_{E}$ as the number of the encoded packets received by the nodes D and E, respectively, after the source stops its transmission. Then, the outage probability (OP) at the destination is formulated by

(14) $$\begin{array}{c} \mathrm{OP}=\mathrm{Pr}\left(N_{D}<H|N_{S}=N_{\mathrm{th}}\right). \end{array}$$

Next, the probability that the source-destination transmission is successful and secure (SS) is defined as

(15) $$\begin{array}{c} \mathrm{SS}=\mathrm{Pr}\left(N_{D}=H,N_{E}<H|N_{S}\leq N_{\mathrm{th}}\right). \end{array}$$

Equation (15) implies that the destination can receive sufficient number of the encoded packets $\left(N_{D}=H\right)$ before the eavesdropper $\left(N_{E}<H\right)$ when the number of time slots used is less than or equal to $N_{\mathrm{th}}$ $\left(N_{S}\leq N_{\mathrm{th}}\right)$.

Let us consider the intercept probability (IP) defined as the probability that the eavesdropper can obtain *H* encoded packets before or at same time with the destination:

(16) $$\begin{array}{c} \mathrm{IP}=\mathrm{Pr}\left(N_{E}=H,N_{D}\leq H|N_{S}\leq N_{\mathrm{th}}\right). \end{array}$$

We note from (16) that when the eavesdropper obtains *H* encoded packets, it does not need to receive more encoded packets, regardless of whether the source will transmit the encoded packets in the next time slots. Instead, it will start to decode the original data of the source. Finally, we study the average number of the time slots used to transmit encoded packets to the destination, which can be formulated by

(17) $$\begin{array}{c} \mathrm{TS}=\sum_{v=0}^{H-1}N_{\mathrm{th}}\mathrm{Pr}\left(N_{D}=v|N_{S}=N_{\mathrm{th}}\right)+\sum_{t=H}^{N_{\mathrm{th}}}t\mathrm{Pr}\left(N_{D}=H|N_{S}=t\right). \end{array}$$

In (17), $\mathrm{Pr}\left(N_{D}=v|N_{S}=N_{\mathrm{th}}\right)$ is the probability that the number of the encoded packets received at the destination is $v\left(0\leq v<H\right)$ when the source used $N_{\mathrm{th}}$ time slots (the destination is in outage), and $\mathrm{Pr}\left(N_{D}=H|N_{S}=t\right)$ is the probability that D can obtain sufficient number of the encoded packets within *t* time slots, where $H\leq t\leq N_{\mathrm{th}}$ (the data transmission is successful).

## 3. Performance Analysis

### 3.1. Derivations of $\rho_{D}$ and $\rho_{E}$

**Proposition** **1.** *If $1-\kappa_{D}^{2}\theta_{\mathrm{th}}\leq 0$, then $\rho_{D}=1$, and if $1-\kappa_{D}^{2}\theta_{\mathrm{th}}>0,$$\rho_{D}$ can be expressed by an exact closed-form formula as*

(18) $$\begin{array}{cc} \rho_{D}=1+ & \sum_{m=1}^{M}{\left(-1\right)}^{m}C_{M}^{m}\exp\left(-m\lambda_{\mathrm{SD}}\omega_{0}\right)\prod_{k=1}^{K}\frac{\lambda_{I_{k}D}}{\lambda_{I_{k}D}+m\lambda_{\mathrm{SD}}\omega_{k}}. \end{array}$$

**Proof.**  See the proof and notations in Appendix A. □

**Proposition** **2.** *If $1-\kappa_{E}^{2}\theta_{\mathrm{th}}\leq 0$, then $\rho_{E}=1$, and if $1-\kappa_{E}^{2}\theta_{\mathrm{th}}>0,$ we can obtain an exact closed-form expression of $\rho_{E}$ as*

(19) $$\begin{array}{cc} \rho_{E}=1 & -\left(\prod_{k=1}^{K}\frac{\lambda_{I_{k}E}}{\lambda_{I_{k}E}+\lambda_{\mathrm{SE}}\vartheta_{k}}\right)\exp\left(-\lambda_{\mathrm{SE}}\vartheta_{0}\right)\\ & \times \;\left[\frac{\lambda_{\mathrm{JE}}\Omega_{\mathrm{SJ}}}{\lambda_{\mathrm{SE}}}\beta_{0}\exp\;\left(\frac{\lambda_{\mathrm{JE}}\Omega_{\mathrm{SJ}}}{\lambda_{\mathrm{SE}}}\right)\;E_{1}\left(\frac{\lambda_{\mathrm{JE}}\Omega_{\mathrm{SJ}}}{\lambda_{\mathrm{SE}}}\right)\;+\;\sum_{k=1}^{K}\frac{\lambda_{\mathrm{JE}}\Omega_{I_{k}J}}{\lambda_{\mathrm{SE}}}\beta_{k}\exp\;\left(\frac{\lambda_{\mathrm{JE}}\Omega_{I_{k}J}}{\lambda_{\mathrm{SE}}}\right)\;E_{1}\left(\frac{\lambda_{\mathrm{JE}}\Omega_{I_{k}J}}{\lambda_{\mathrm{SE}}}\right)\right]\;.\; \end{array}$$

**Proof.**  See the proof and notations in Appendix B. □

In case where α=0, Equation (19) reduces to

(20) $$\begin{array}{cc} \rho_{E}=1 & -\left(\prod_{k=1}^{K}\frac{\lambda_{I_{k}E}}{\lambda_{I_{k}E}+\lambda_{\mathrm{SE}}\vartheta_{k}}\right)\exp\left(-\lambda_{\mathrm{SE}}\vartheta_{0}\right). \end{array}$$

### 3.2. Analysis of Outage Probability (OP)

As defined in (14), an exact closed-form expression of OP can be provided as follows:

(21) $$\begin{array}{c} \mathrm{OP}=\sum_{N_{D}=0}^{H-1}C_{N_{\mathrm{th}}}^{N_{D}}{\left(1-\rho_{D}\right)}^{N_{D}}{\left(\rho_{D}\right)}^{N_{\mathrm{th}}-N_{D}}. \end{array}$$

It is noted from (21) that the possible values of $N_{D}$ are from 0 to H−1, and there are $C_{N_{\mathrm{th}}}^{N_{D}}$ possible cases for each value of $N_{D}$.

### 3.3. Analysis of Successful and Secure Communication (SS)

From (15), we can rewrite SS by

(22) $$\begin{array}{cc} \mathrm{SS} & =\sum_{u=H}^{N_{\mathrm{th}}}\mathrm{Pr}\left(N_{D}=H|N_{S}=u\right)\times \sum_{t=0}^{H-1}\mathrm{Pr}\left(N_{E}=t|N_{S}=u\right). \end{array}$$

In (22), $\mathrm{Pr}\left(N_{D}=H|N_{S}=u\right)$ is the probability that the destination can correctly receive *H* encoded packets when the number of time slots used is *u*. Since the data transmission between the source and the destination ends in the u−th time slot, $\mathrm{Pr}\left(N_{D}=H|N_{S}=u\right)$ is given as

(23) $$\begin{array}{c} \mathrm{Pr}\left(N_{D}=H|N_{S}=u\right)=C_{u-1}^{u-H}{\left(1-\rho_{D}\right)}^{H}{\left(\rho_{D}\right)}^{u-H}. \end{array}$$

Moreover, $\mathrm{Pr}\left(N_{E}=t|N_{S}=u\right)$ in (22) presents the probability that the number of encoded packets obtained at the eavesdropper is *t*. Similar to (21), we have

(24) $$\begin{array}{c} \mathrm{Pr}\left(N_{E}=t|N_{S}=u\right)=C_{u}^{t}{\left(1-\rho_{E}\right)}^{t}{\left(\rho_{E}\right)}^{u-t}. \end{array}$$

Substituting (23) and (24) into (22), an exact closed-form expression of SS can be given as

(25) $$\begin{array}{c} \mathrm{SS}=\sum_{u=H}^{N_{\mathrm{th}}}C_{u-1}^{u-H}{\left(1-\rho_{D}\right)}^{H}{\left(\rho_{D}\right)}^{u-H}\times \sum_{t=0}^{H-1}C_{u}^{t}{\left(1-\rho_{E}\right)}^{t}{\left(\rho_{E}\right)}^{u-t}. \end{array}$$

### 3.4. Analysis of Intercept Probability (IP)

The intercept probability (IP) in (16) is given by

(26) $$\begin{array}{c} \mathrm{IP}=\sum_{u=H}^{N_{\mathrm{th}}}\mathrm{Pr}\left(N_{E}=H|N_{S}=u\right)\times \left[\mathrm{Pr}\left(N_{D}=H|N_{S}=u\right)+\sum_{v=0}^{H-1}\mathrm{Pr}\left(N_{D}=v|N_{S}=u\right)\right]. \end{array}$$

In (26), $\mathrm{Pr}\left(N_{E}=H|N_{S}=u\right)$ is the probability that the eavesdropper can receive sufficient number of the encoded packets in *u* time slots, which can be calculated similarly to (23) as

(27) $$\begin{array}{c} \mathrm{Pr}\left(N_{E}=H|N_{S}=u\right)=C_{u-1}^{u-H}{\left(1-\rho_{E}\right)}^{H}{\left(\rho_{E}\right)}^{u-H}. \end{array}$$

Next, $\mathrm{Pr}\left(N_{D}=H|N_{S}=u\right)$ in (26) is calculated by (23), and $\mathrm{Pr}\left(N_{D}=v|N_{S}=u\right)$ in (26) can be obtained by

(28) $$\begin{array}{c} \mathrm{Pr}\left(N_{D}=v|N_{S}=u\right)=C_{u}^{v}{\left(1-\rho_{D}\right)}^{v}{\left(\rho_{D}\right)}^{u-v}. \end{array}$$

Plugging (23), (26), (27) and (28) together, we obtain

(29) $$\begin{array}{c} \mathrm{IP}=\sum_{u=H}^{N_{\mathrm{th}}}C_{u-1}^{u-H}{\left(1-\rho_{E}\right)}^{H}{\left(\rho_{E}\right)}^{u-H}\times \left[C_{u-1}^{u-H}{\left(1-\rho_{D}\right)}^{H}{\left(\rho_{D}\right)}^{u-H}+\sum_{v=0}^{H-1}C_{u}^{v}{\left(1-\rho_{D}\right)}^{v}{\left(\rho_{D}\right)}^{u-v}\right]. \end{array}$$

### 3.5. Analysis of Average Number of Time Slots (TS)

Similarly, the probability $\mathrm{Pr}\left(N_{D}=v|N_{S}=N_{\mathrm{th}}\right)$ and $\mathrm{Pr}\left(N_{D}=H|N_{S}=t\right)$ in (17) can be calculated respectively as

(30) $$\begin{array}{c} \mathrm{Pr}\left(N_{D}=v|N_{S}=N_{\mathrm{th}}\right)=C_{N_{\mathrm{th}}}^{v}{\left(1-\rho_{D}\right)}^{v}{\left(\rho_{D}\right)}^{N_{\mathrm{th}}-H},\\ \mathrm{Pr}\left(N_{D}=H|N_{S}=t\right)=C_{t-1}^{t-H}{\left(1-\rho_{D}\right)}^{H}{\left(\rho_{D}\right)}^{t-H}. \end{array}$$

Substituting (30) into (17), we obtain an exact closed-form formula for the average number of time slots used by the source as

(31) $$\begin{array}{c} \mathrm{TS}=N_{\mathrm{th}}\sum_{v=0}^{H-1}C_{N_{\mathrm{th}}}^{v}{\left(1-\rho_{D}\right)}^{v}{\left(\rho_{D}\right)}^{N_{\mathrm{th}}-v}+\sum_{t=H}^{N_{\mathrm{th}}}tC_{t-1}^{t-H}{\left(1-\rho_{D}\right)}^{H}{\left(\rho_{D}\right)}^{t-H}. \end{array}$$

## 4. Simulation Results

In this section, Monte Carlo simulations are presented to verify the theoretical results. For illustration purpose, in all of the simulations, we fix the required number of the encoded packets by 10 $\left(H=10\right)$, the energy conversion efficiency by 1 $\left(\eta =1\right)$, the total block time by 1 $\left(T=1\right)$, the number of the interference sources by 3 $\left(K=3\right)$, the parameters of the interference links by $\lambda_{I_{1}D}=\lambda_{I_{1}E}=\lambda_{I_{1}J}=3$, $\lambda_{I_{2}D}=\lambda_{I_{2}E}=\lambda_{I_{2}J}=4$ and $\lambda_{I_{3}D}=\lambda_{I_{3}E}=\lambda_{I_{3}J}=5$, and the parameters of the remaining links by 1 $\left(\lambda_{\mathrm{SD}}=\lambda_{\mathrm{SE}}=\lambda_{\mathrm{SJ}}=\lambda_{\mathrm{JE}}=1\right)$. In the figures, the simulation and theoretical results are denoted by Sim and Theo, respectively.

**Figure 2:**
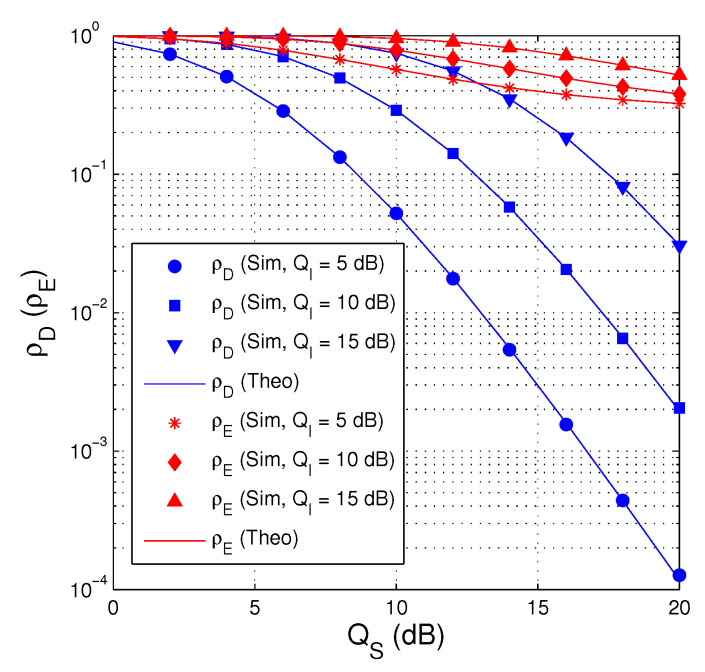
$\rho_{D}$ and $\rho_{E}$ as a function of $Q_{S}$ in dB when M=3, α=0.3, $\kappa_{D}^{2}=\kappa_{E}^{2}=0.1$ and $C_{\mathrm{th}}=0.75$.

In Figure 2, we present the probability $\rho_{D}$ and $\rho_{E}$ as a function of $Q_{S}$ in dB. In this figure, the number of antenna equipped by the source is set to 3 $\left(M=3\right)$, the fraction of time allocated for the EH phase is fixed by 0.3 $\left(\alpha =0.3\right)$, the hardware impairment levels are assigned by $\kappa_{D}^{2}=\kappa_{E}^{2}=0.1$, and the target rate is set to 0.75 $\left(C_{\mathrm{th}}=0.75\right)$. It can be seen from Figure 2 that $\rho_{D}$ and $\rho_{E}$ decrease with the increasing of $Q_{S}$ and the decreasing of $Q_{I}$. However, $\rho_{D}$ is much smaller than $\rho_{E}$ at medium and high $Q_{S}$ regimes. We also obverse that the simulation and theoretical results are in good agreement, which validates our derivations.

**Figure 3:**
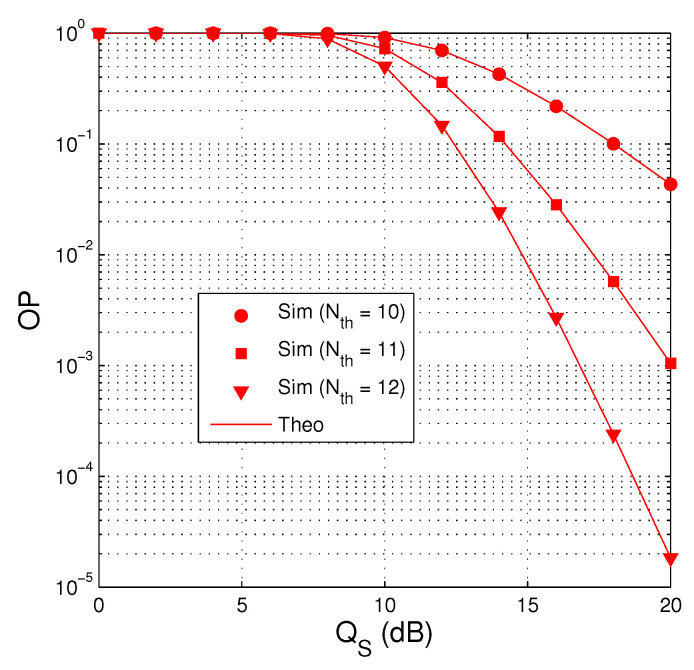
OP as a function of $Q_{S}$ in dB when $Q_{I}=7.5$ dB, M=2, α=0.1, $\kappa_{D}^{2}=\kappa_{E}^{2}=0$ and $C_{\mathrm{th}}=1$.

Figure 3 presents outage performance of the proposed protocol as a function of $Q_{S}$ in dB with $Q_{I}=7.5$ dB, M=2, α=0.1, $\kappa_{D}^{2}=\kappa_{E}^{2}=0$ and $C_{\mathrm{th}}=1$. It is shown in Figure 3 that the impact of the co-channel interference on the performance is negative, i.e., the value of OP is very high at low $Q_{S}$ regimes. In particularly, when the value of $Q_{S}$ is lower than that of $Q_{I}$, OP is almost equal to 1. We can also observe that the outage performance is better with high value of $N_{\mathrm{th}}$ because the source has more time slots to transmit the encoded packets to the destination.

**Figure 4:**
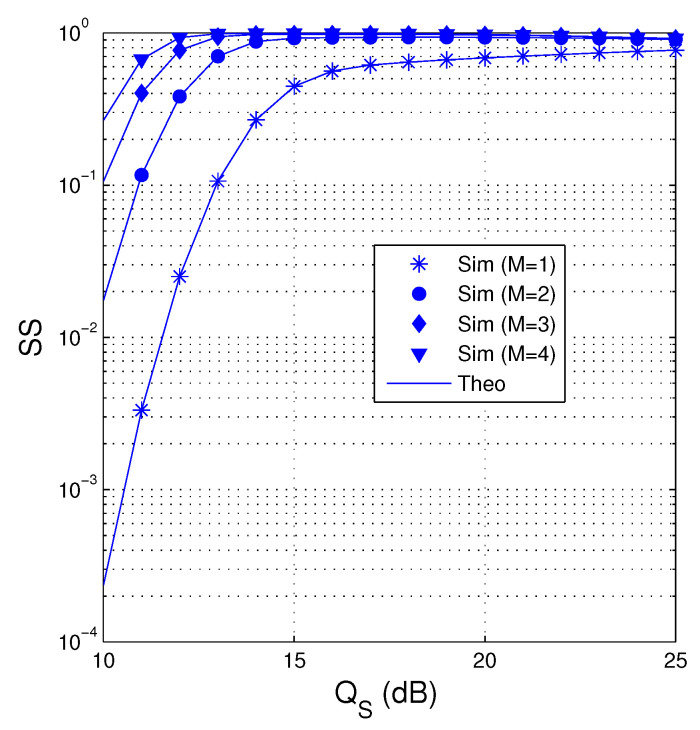
SS as a function of $Q_{S}$ in dB when $Q_{I}=10$ dB, α=0.1, $\kappa_{D}^{2}=0.1$, $\kappa_{D}^{2}=0$, $N_{\mathrm{th}}=20$ and $C_{\mathrm{th}}=1.5$.

In Figure 4, we present the value of SS as a function of $Q_{S}$ in dB when $Q_{I}=10$ dB , α=0.1, $\kappa_{D}^{2}=0.1$, $\kappa_{E}^{2}=0$, $N_{\mathrm{th}}=20$ and $C_{\mathrm{th}}=1.5$. We can see that the proposed protocol obtains higher value of SS when more antennas are equipped at the source. It is also seen that when M=1, the SS performance is significantly degraded because no transmit diversity gain is obtained. Moreover, SS also increases as increasing $Q_{S}$. It is due to the fact that at high $Q_{S}$ values, the destination almost obtains sufficient number of the encoded packets before the eavesdropper. However, it can be seen from Figure 4 that when the value of $Q_{S}$ is very high, the value of SS slightly decreases due to high overhearing possibility of the eavesdropper. Moreover, the SS performance in all values of M(M>1) is almost same at high $Q_{S}$ regimes.

**Figure 5:**
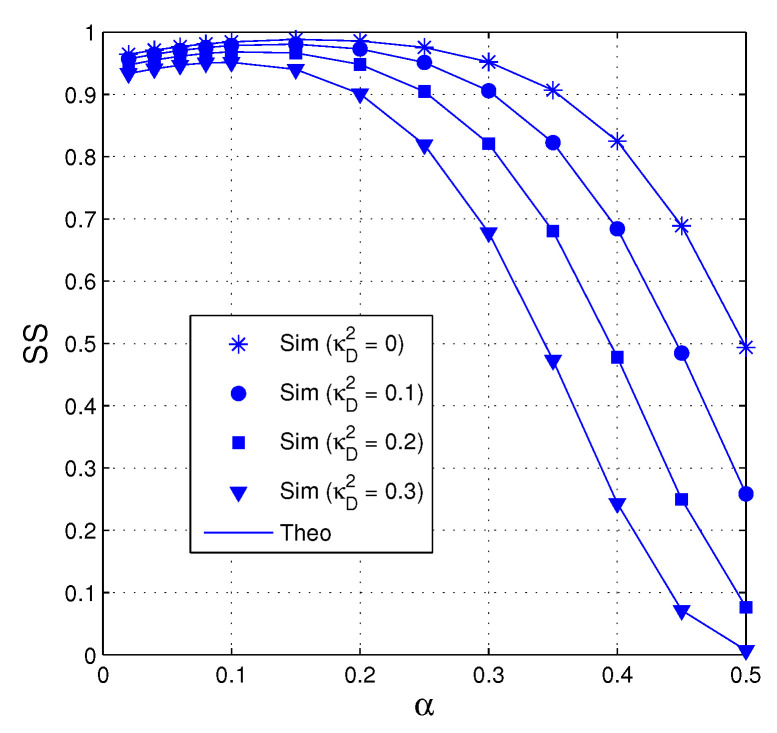
SS as a function of α when $Q_{S}=Q_{I}=15$ dB, M=3, $\kappa_{E}^{2}=0.1$, $N_{\mathrm{th}}=15$ and $C_{\mathrm{th}}=0.7$.

In Figure 5, the value of SS is presented as a function of α when $Q_{S}=Q_{I}=15$ dB, M=3, $\kappa_{E}^{2}=0.1$, $N_{\mathrm{th}}=15$ and $C_{\mathrm{th}}=0.7$. As we can see, the performance significantly degrades with high hardware impairment levels of the data links, i.e., $\kappa_{D}^{2}$ is high. Moreover, we can observe from that the fraction of time allocated for the EH phase impacts on the value of SS. It can be seen that there exists an optimal value of α at which the value of SS is highest.

**Figure 6:**
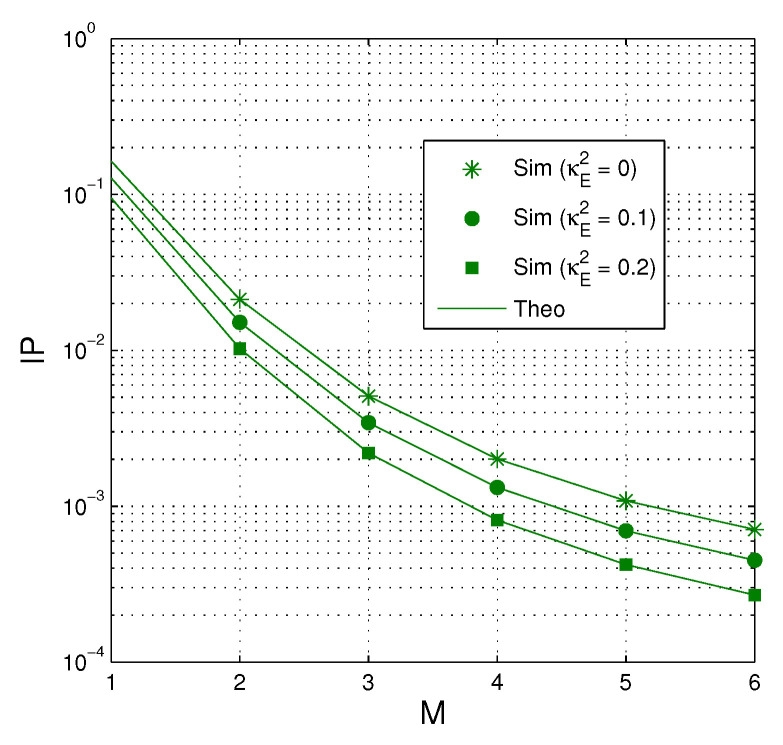
IP as a function of *M* when $Q_{S}=Q_{I}=20$ dB, $\kappa_{D}^{2}=0.2$, α=0.3, $N_{\mathrm{th}}=20$ and $C_{\mathrm{th}}=0.5$.

In Figure 6, the intercept probability of the proposed protocol is presented as a function of *M* when $Q_{S}=Q_{I}=20$ dB, $\kappa_{D}^{2}=0.2$, α=0.3, $N_{\mathrm{th}}=20$ and $C_{\mathrm{th}}=0.5$. As we can see, the value of IP decreases when more antennas are equipped at the source. Also, IP is lower when the hardware impairment level of the eavesdropping links is high.

**Figure 7:**
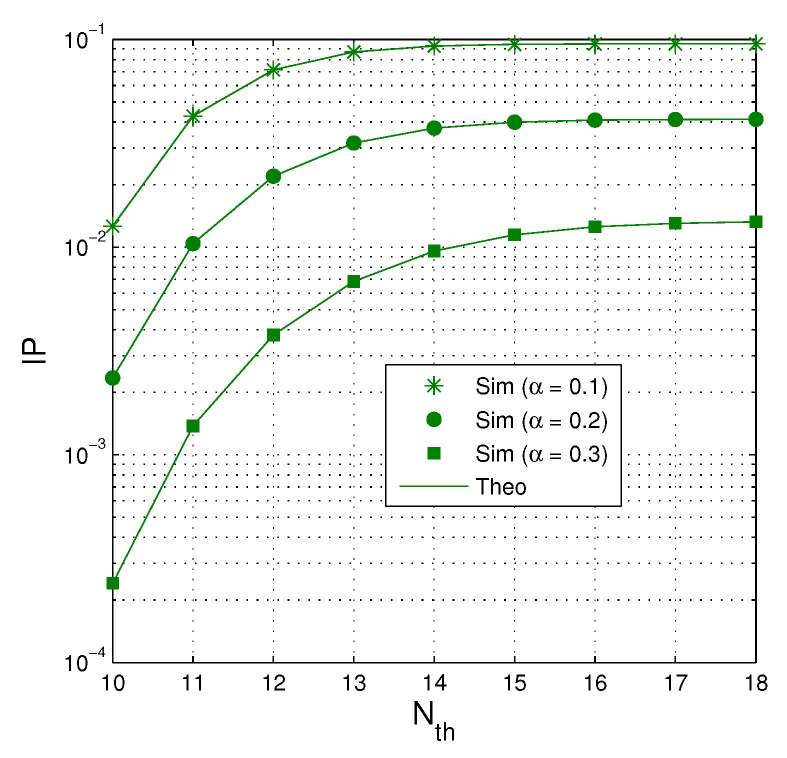
IP as a function of $N_{\mathrm{th}}$ when $Q_{S}=Q_{I}=20$ dB, M=2, $\kappa_{D}^{2}=\kappa_{E}^{2}=0$ and $C_{\mathrm{th}}=0.5$.

Figure 7 investigates impact of $N_{\mathrm{th}}$ on the intercept probability as $Q_{S}=Q_{I}=20$ dB, M=2, $\kappa_{D}^{2}=\kappa_{E}^{2}=0$, and $C_{\mathrm{th}}=0.5$. It can be seen that the value of IP is higher when the number of $N_{\mathrm{th}}$ increases. However, when the number of $N_{\mathrm{th}}$ is high enough, IP converges to a constant. As expected, IP is lower when more time used for the EH phase (because the transmit power of the jammer is higher).

**Figure 8:**
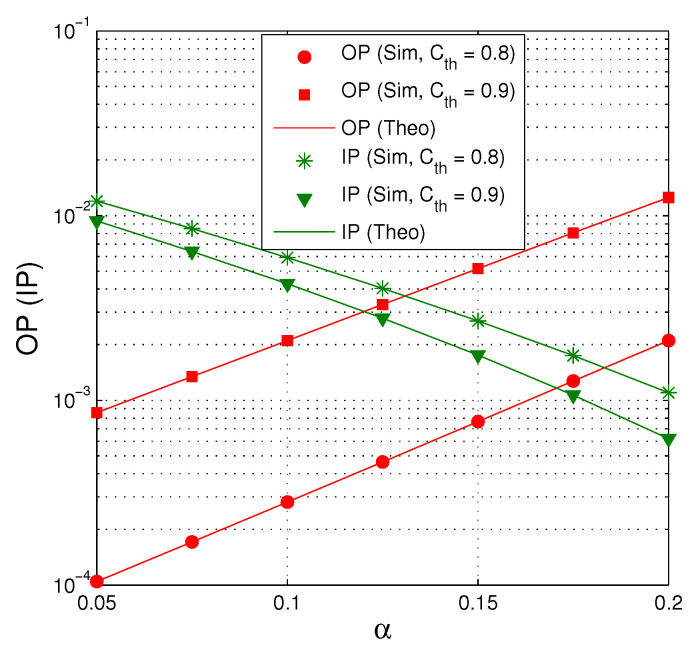
OP and IP as a function of α when $Q_{S}=Q_{I}=15$ dB, M=4, $\kappa_{D}^{2}=\kappa_{E}^{2}=0$ and $N_{\mathrm{th}}=16$.

Figure 8 presents OP and IP as a function of α when $Q_{S}=Q_{I}=15$ dB, M=4, $\kappa_{D}^{2}=\kappa_{E}^{2}=0$ and $N_{\mathrm{th}}=16$. We can see that there exists a trade-off between OP and IP. Indeed, OP increases when increasing the value of α, while IP decreases with higher value of α. We can also see that when $C_{\mathrm{th}}=0.8$, OP is below $10^{-3}$ when the value of α is higher than (about) 0.15, but the intercept probability is higher than $2.5\times 10^{-3}$. In addition, OP significantly decreases as decreasing the value of $C_{\mathrm{th}}$.

**Figure 9:**
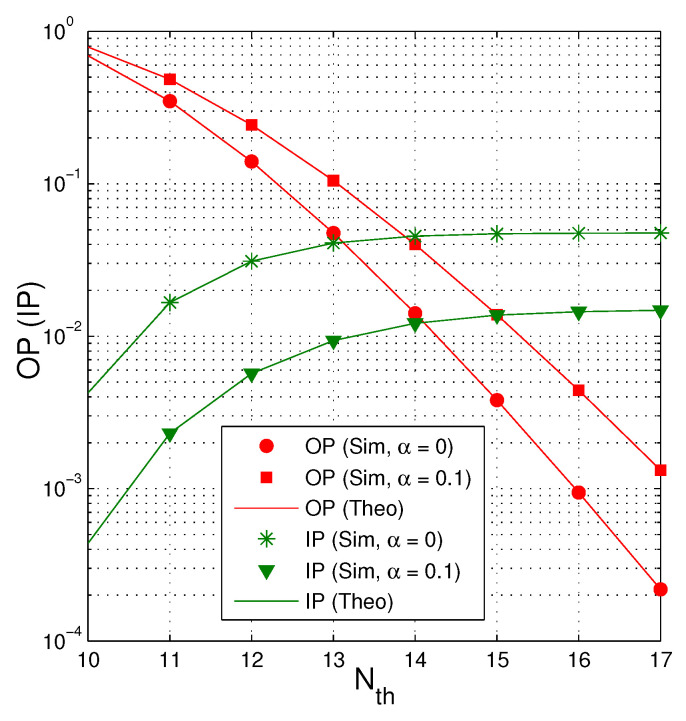
OP and IP as a function of $N_{\mathrm{th}}$ when $Q_{S}=Q_{I}=15$ dB, M=3, $\kappa_{D}^{2}=\kappa_{E}^{2}=0.1$ and $C_{\mathrm{th}}=0.75$.

Figure 9 shows the trade-off between OP and IP when $Q_{S}=Q_{I}=15$ dB, M=3, $\kappa_{D}^{2}=\kappa_{E}^{2}=0.1$ and $C_{\mathrm{th}}=0.75$. As we can observe, when the cooperative jamming technique is not used $\left(\alpha =0\right)$, the OP value is lower but the IP one is higher. Similarly, to increase the reliability of the data transmission, we can increase the number of $N_{\mathrm{th}}$. However, the intercept possibility of the eavesdropper also increases with higher value of $N_{\mathrm{th}}$.

**Figure 10:**
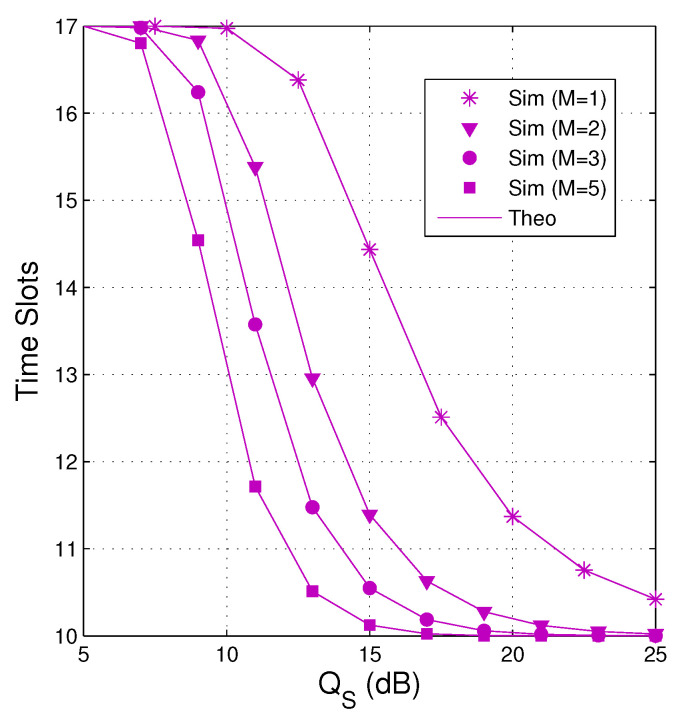
Average number of time slots as a function of $Q_{S}$ in dB when $Q_{I}=10$ dB, α=0.2, $\kappa_{D}^{2}=\kappa_{E}^{2}=0.05$, $N_{\mathrm{th}}=17$ and $C_{\mathrm{th}}=1$.

In Figure 10, we present average number of the time slots as a function of $Q_{S}$ in dB when $Q_{I}\;=\;10$ dB, α=0.2, $\kappa_{D}^{2}=\kappa_{E}^{2}=0.05$, $N_{\mathrm{th}}=17$ and $C_{\mathrm{th}}=1$. We see that the number of time slots used decreases when increasing the number of antennas and the transmit power of the source. It is also seen that as the source has a single antenna (M=1), the average number of time slots is much higher. Moreover, reducing the number of time slots means reducing the delay time and transmit power, which are an important metric of wireless communication systems.

From Figure 3, Figure 4, Figure 5, Figure 6, Figure 7, Figure 8, Figure 9 and Figure 10, it is worth noting that the theoretical results and simulation results are in good agreement which validates the theoretical derivations.

## 5. Conclusions

In this paper, we proposed an FC-based MISO scheme using the TAS and EH-based cooperative jamming techniques for the secure communication under the joint impact of hardware impairments and co-channel interference. The performance of the proposed scheme such as outage probability (OP), probability of successful and secure communication (SS), intercept probability (IP) and average number of the time slots was evaluated via both simulation and theory. The results presented that the hardware impairment levels, the co-channel interference, the fraction of time allocated for the EH phase and the number of transmit antennas at the source significantly impact on the system performance. Moreover, there exists a trade-off between the security and reliability, i.e., between OP and IP. Finally, the fraction of time allocated for the EH phase should be designed appropriately to optimize system performance.

## Appendix A. Proof of Proposition 1

From (7) and (11), we obtain

(A1) $$\begin{array}{cc} \rho_{D} & =\mathrm{Pr}\left(\frac{Q_{S}\gamma_{S_{b}D}}{\kappa_{D}^{2}Q_{S}\gamma_{S_{b}D}+\sum_{k=1}^{K}Q_{I_{k}}\gamma_{I_{k}D}+1}<\theta_{\mathrm{th}}\right)\\ & =\mathrm{Pr}\left(\left(1-\kappa_{D}^{2}\theta_{\mathrm{th}}\right)Q_{S}\gamma_{S_{b}D}<\sum_{k=1}^{K}Q_{I_{k}}\theta_{\mathrm{th}}\gamma_{I_{k}D}+\theta_{\mathrm{th}}\right). \end{array}$$

From (A1), we observe that if $1-\kappa_{D}^{2}\theta_{\mathrm{th}}\leq 0$, then $\rho_{D}=1$; and if $1-\kappa_{D}^{2}\theta_{\mathrm{th}}>0$, we can rewrite (A1) as

(A2) $$\begin{array}{c} \rho_{D}=\mathrm{Pr}\left(\gamma_{S_{b}D}<\sum_{k=1}^{K}\omega_{k}\gamma_{I_{k}D}+\omega_{0}\right), \end{array}$$

where

(A3) $$\begin{array}{c} \omega_{0}=\frac{\theta_{\mathrm{th}}}{\left(1-\kappa_{D}^{2}\theta_{\mathrm{th}}\right)Q_{S}},\;\omega_{k}=\frac{\theta_{\mathrm{th}}Q_{I_{k}}}{\left(1-\kappa_{D}^{2}\theta_{\mathrm{th}}\right)Q_{S}}. \end{array}$$

Moreover, Equation (A2) can be rewritten by

(A4) $$\begin{array}{c} \rho_{D}=\int_{0}^{+\infty }\ldots \int_{0}^{+\infty }F_{\gamma_{S_{b}D}}\left(\sum_{k=1}^{K}\omega_{k}x_{k}+\omega_{0}\right)f_{\gamma_{I_{1}D}}\left(x_{1}\right)\ldots f_{\gamma_{I_{K}D}}\left(x_{K}\right)dx_{1}\ldots dx_{K}. \end{array}$$

Using the CDF obtained by (3), we have

(A5) $$\begin{array}{c} F_{\gamma_{S_{b}D}}\left(\sum_{k=1}^{K}\omega_{k}x_{k}+\omega_{0}\right)=1+\sum_{m=1}^{M}{\left(-1\right)}^{m}C_{M}^{m}\exp\left(-m\lambda_{\mathrm{SD}}\omega_{0}\right)\exp\left(-m\lambda_{\mathrm{SD}}\sum_{k=1}^{K}\omega_{k}x_{k}\right). \end{array}$$

Substituting (A5) and the PDF of $\gamma_{I_{k}D}$ in (1); after some manipulations, we can obtain (18), and finish the proof here.

## Appendix B. Proof of Proposition 2

Combining (9) and (13), we have

(A6) $$\begin{array}{c} \rho_{E}=\mathrm{Pr}\left(\left(1-\kappa_{E}^{2}\theta_{\mathrm{th}}\right)Q_{S}\gamma_{S_{b}E}<\chi \theta_{\mathrm{th}}\left(Q_{S}\gamma_{S_{b}J}+\sum_{k=1}^{K}Q_{I_{k}}\gamma_{I_{k}J}\right)\gamma_{\mathrm{JE}}+\theta_{\mathrm{th}}\sum_{k=1}^{K}Q_{I_{k}}\gamma_{I_{k}E}+\theta_{\mathrm{th}}\right). \end{array}$$

We observe from (A6) that if $1-\kappa_{E}^{2}\theta_{\mathrm{th}}\leq 0$, then $\rho_{E}=1$, and if $1-\kappa_{E}^{2}\theta_{\mathrm{th}}>0$, we can rewrite (A6) as

(A7) $$\begin{array}{c} \rho_{E}=\mathrm{Pr}\left(\gamma_{S_{b}E}<\vartheta_{0}+\sum_{k=1}^{K}\vartheta_{k}\gamma_{I_{k}E}+\left(\mu_{0}\gamma_{S_{b}J}+\sum_{k=1}^{K}\mu_{k}\gamma_{I_{k}J}\right)\gamma_{\mathrm{JE}}\right), \end{array}$$

where

(A8) $$\begin{array}{c} \vartheta_{0}=\frac{\theta_{\mathrm{th}}}{\left(1-\kappa_{E}^{2}\theta_{\mathrm{th}}\right)Q_{S}},\;\;\vartheta_{k}=\frac{\theta_{\mathrm{th}}Q_{I_{k}}}{\left(1-\kappa_{E}^{2}\theta_{\mathrm{th}}\right)Q_{S}},\mu_{0}=\frac{\chi \theta_{\mathrm{th}}}{1-\kappa_{E}^{2}\theta_{\mathrm{th}}},\;\;\mu_{k}=\frac{\chi \theta_{\mathrm{th}}Q_{I_{k}}}{\left(1-\kappa_{E}^{2}\theta_{\mathrm{th}}\right)Q_{S}}. \end{array}$$

Setting $Z=\left(\mu_{0}\gamma_{S_{b}J}+\sum_{k=1}^{K}\mu_{k}\gamma_{I_{k}J}\right)\gamma_{\mathrm{JE}}$, from (A7), we have

(A9) $$\begin{array}{c} \rho_{E}=\int_{0}^{+\infty }\ldots \int_{0}^{+\infty }\left[\begin{array}{c} F_{\gamma_{S_{b}E}}\left(\vartheta_{0}+\sum_{k=1}^{K}\vartheta_{k}x_{k}+z\right)f_{\gamma_{I_{1}E}}\left(x_{1}\right)\ldots f_{\gamma_{I_{K}E}}\left(x_{K}\right)f_{Z}\left(z\right) \end{array}\right]dx_{1}\ldots dx_{K}dz. \end{array}$$

Substituting the CDF of $\gamma_{S_{b}E}$ and the PDF of $\gamma_{I_{k}D}$ provided by (1) into (A9), after some manipulations, which yields

(A10) $$\begin{array}{cc} \rho_{E}=1 & -\left(\prod_{k=1}^{K}\frac{\lambda_{I_{k}E}}{\lambda_{I_{k}E}+\lambda_{\mathrm{SE}}\vartheta_{k}}\right)\exp\left(-\lambda_{\mathrm{SE}}\vartheta_{0}\right)\times \underset{I}{\underset{︸}{\int_{0}^{+\infty }\exp\left(-\lambda_{\mathrm{SE}}z\right)f_{Z}\left(z\right)dz}}. \end{array}$$

Now, our objective is to calculate the integral I in (A10). At first, we rewrite I under the following form:

(A11) $$\begin{array}{cc} I & =\int_{0}^{+\infty }\exp\left(-\lambda_{\mathrm{SE}}z\right)f_{Z}\left(z\right)dz\\ & =\int_{0}^{+\infty }\lambda_{\mathrm{SE}}\exp\left(-\lambda_{\mathrm{SE}}z\right)F_{Z}\left(z\right)dz. \end{array}$$

Next, we attempt to find the CDF of *Z*. Setting $Y=\mu_{0}\gamma_{S_{b}J}+\sum_{k=1}^{K}\mu_{k}\gamma_{I_{k}J}$, the CDF of *Z* can be formulated by

(A12) $$\begin{array}{cc} F_{Z}\left(z\right) & =\mathrm{Pr}\left(Y\gamma_{\mathrm{JE}}<z\right)\\ & =\int_{0}^{+\infty }F_{Y}\left(\frac{z}{x}\right)\lambda_{\mathrm{JE}}\exp\left(-\lambda_{\mathrm{JE}}x\right)dx. \end{array}$$

Before calculating the CDF of *Y*, we note that *Y* is sum of the exponential RVs, i.e., $\mu_{0}\gamma_{S_{b}J}$ and $\mu_{k}\gamma_{I_{k}J}$. Indeed, because $\gamma_{S_{b}J}$ and $\gamma_{I_{k}J}$ are exponential RVs whose parameters are $\lambda_{\mathrm{SJ}}$ and $\lambda_{I_{k}J}$, respectively, hence $\mu_{0}\gamma_{S_{b}J}$ and $\mu_{k}\gamma_{I_{k}J}$ are also exponential RVs, and their parameters are $\lambda_{\mathrm{SJ}}/\mu_{0}$ and $\lambda_{I_{k}J}/\mu_{k}$, respectively. Hence, the CDF of *Y* can be given as

(A13) $$\begin{array}{c} \;F_{Y}\left(y\right)\;=\;1\;-\beta_{0}\exp\left(-\Omega_{\mathrm{SJ}}y\right)\;-\;\sum_{k=1}^{K}\beta_{k}\exp\left(-\Omega_{I_{k}J}y\right), \end{array}$$

where

(A14) $$\begin{array}{cc} \Omega_{\mathrm{SJ}}=\frac{\lambda_{\mathrm{SJ}}}{\mu_{0}},\;\Omega_{I_{k}J}=\frac{\lambda_{I_{k}J}}{\mu_{k}},\beta_{0}=\prod_{k=1}^{K}\frac{\Omega_{I_{k}J}}{\Omega_{I_{k}J}-\Omega_{\mathrm{SJ}}},\beta_{k} & =\frac{\Omega_{\mathrm{SJ}}}{\Omega_{\mathrm{SJ}}-\Omega_{I_{k}J}}\prod_{t=1,t\neq k}^{K}\frac{\Omega_{I_{t}J}}{\Omega_{I_{t}J}-\Omega_{I_{k}J}}. \end{array}$$

Substituting (A13) into (A12), we obtain

(A15) $$\begin{array}{cc} F_{Z}\left(z\right) & =1-\beta_{0}\int_{0}^{+\infty }\lambda_{\mathrm{JE}}\exp\left(-\lambda_{\mathrm{JE}}x\right)\exp\left(-\Omega_{\mathrm{SJ}}\frac{z}{x}\right)dy\\ & -\sum_{k=1}^{K}\beta_{k}\;\;\int_{0}^{+\infty }\;\lambda_{\mathrm{JE}}\exp\left(-\lambda_{\mathrm{JE}}x\right)\exp\left(-\Omega_{I_{k}J}\frac{z}{x}\right)dy. \end{array}$$

Using (Equation (3.324.1) of [45]) for the corresponding integrals in (A15), we arrive at

(A16) $$\begin{array}{cc} F_{Z}\left(z\right) & =1-2\beta_{0}\sqrt{\lambda_{\mathrm{JE}}\Omega_{\mathrm{SJ}}z}K_{1}\left(2\sqrt{\lambda_{\mathrm{JE}}\Omega_{\mathrm{SJ}}z}\right)\\ & -\sum_{k=1}^{K}2\beta_{k}\sqrt{\lambda_{\mathrm{JE}}\Omega_{I_{k}J}z}K_{1}\left(2\sqrt{\lambda_{\mathrm{JE}}\Omega_{I_{k}J}z}\right), \end{array}$$

where $K_{1}\left(.\right)$ is modified Bessel function of the second kind [45]. Then, substituting (A16) into (A11), we obtain (A17) as

(A17) $$\begin{array}{cc} I & =1-2\beta_{0}\int_{0}^{+\infty }\lambda_{\mathrm{SE}}\exp\left(-\lambda_{\mathrm{SE}}z\right)\sqrt{\lambda_{\mathrm{JE}}\Omega_{\mathrm{SJ}}z}K_{1}\left(2\sqrt{\lambda_{\mathrm{JE}}\Omega_{\mathrm{SJ}}z}\right)dz\\ & -\sum_{k=1}^{K}2\beta_{k}\int_{0}^{+\infty }\lambda_{\mathrm{SE}}\exp\left(-\lambda_{\mathrm{SE}}z\right)\sqrt{\lambda_{\mathrm{JE}}\Omega_{I_{k}J}z}K_{1}\left(2\sqrt{\lambda_{\mathrm{JE}}\Omega_{I_{k}J}z}\right)dz. \end{array}$$

Next, changing variable $t=\sqrt{z}$, we can rewrite (A17) as

(A18) $$\begin{array}{cc} I & =1-4\lambda_{\mathrm{SE}}\sqrt{\lambda_{\mathrm{JE}}\Omega_{\mathrm{SJ}}}\beta_{0}\int_{0}^{+\infty }t^{2}\exp\left(-\lambda_{\mathrm{SE}}t^{2}\right)K_{1}\left(2\sqrt{\lambda_{\mathrm{JE}}\Omega_{\mathrm{SJ}}}t\right)dt\\ & -\sum_{k=1}^{K}4\lambda_{\mathrm{SE}}\sqrt{\Omega_{I_{k}J}\lambda_{\mathrm{JE}}}\beta_{k}\int_{0}^{+\infty }t^{2}\exp\left(-\lambda_{\mathrm{SE}}t^{2}\right)K_{1}\left(2\sqrt{\lambda_{\mathrm{JE}}\Omega_{I_{k}J}}t\right)dt. \end{array}$$

Applying (Equation (6.631.3) of [45]) for the corresponding integrals in (A18), we obtain

(A19) $$\begin{array}{cc} I & =1-\beta_{0}\exp\left(\frac{\lambda_{\mathrm{JE}}\Omega_{\mathrm{SJ}}}{2\lambda_{\mathrm{SE}}}\right)W_{-1,1/2}\left(\frac{\lambda_{\mathrm{JE}}\Omega_{\mathrm{SJ}}}{\lambda_{\mathrm{SE}}}\right)\\ & -\sum_{k=1}^{K}\beta_{k}\exp\left(\frac{\lambda_{\mathrm{JE}}\Omega_{I_{k}J}}{2\lambda_{\mathrm{SE}}}\right)W_{-1,1/2}\left(\frac{\lambda_{\mathrm{JE}}\Omega_{I_{k}J}}{\lambda_{\mathrm{SE}}}\right), \end{array}$$

where $W_{-1,1/2}\left(.\right)$ is the Whittaker function [45].

Moreover, from (Equation (46) of [46]), we have

(A20) $$\begin{array}{c} \exp\left(\frac{x}{2}\right)W_{-1,1/2}\left(x\right)=1-x\exp\left(x\right)E_{1}\left(x\right), \end{array}$$

where $E_{1}\left(.\right)$ is exponential integral function [45].

Combining (A19) and (A20), after some manipulations, we obtain

(A21) $$\begin{array}{c} I=\frac{\lambda_{\mathrm{JE}}\Omega_{\mathrm{SJ}}}{\lambda_{\mathrm{SE}}}\beta_{0}\exp\left(\frac{\lambda_{\mathrm{JE}}\Omega_{\mathrm{SJ}}}{\lambda_{\mathrm{SE}}}\right)E_{1}\left(\frac{\lambda_{\mathrm{JE}}\Omega_{\mathrm{SJ}}}{\lambda_{\mathrm{SE}}}\right)+\sum_{k=1}^{K}\frac{\lambda_{\mathrm{JE}}\Omega_{I_{k}J}}{\lambda_{\mathrm{SE}}}\beta_{k}\exp\left(\frac{\lambda_{\mathrm{JE}}\Omega_{I_{k}J}}{\lambda_{\mathrm{SE}}}\right)E_{1}\left(\frac{\lambda_{\mathrm{JE}}\Omega_{I_{k}J}}{\lambda_{\mathrm{SE}}}\right). \end{array}$$

It is worth noting that to attain (A21), we used the following equation:

(A22) $$\begin{array}{c} \beta_{0}+\sum_{k=1}^{K}\beta_{k}=1. \end{array}$$

Finally, substituting (A21) into (A10), we obtain (19), and finish the proof.

## References

[1] Wyner A.D. (1975). The Wire-tap Channel. Bell Syst. Tech. J..

[2] Csiszar I., Korner J. (1978). Broadcast Channels with Confidential Messages. IEEE Trans. Inf. Theory.

[3] Liu R., Maric I., Spasojevic P., Yates R.D. (2008). Discrete Memoryless Interference and Broadcast Channels with Conffdential Messages: Secrecy Rate Regions. IEEE Trans. Inf. Theory.

[4] Gopala P.K., Lai L., Gamal H.E. (2008). On the Secrecy Capacity of Fading Channels. IEEE Trans. Inf. Theory.

[5] Zhang J., Duong T.Q., Woods R., Marshall A. (2017). Securing Wireless Communications of the Internet of Things from the Physical Layer, An Overview. Entropy.

[6] Sun L., Du Q. (2018). A Review of Physical Layer Security Techniques for Internet of Things: Challenges and Solutions. Entropy.

[7] Tin P.T., Hung D.T., Tan N.N., Duy T.T., Voznak M. (2019). Secrecy Performance Enhancement for Underlay Cognitive Radio Networks Employing Cooperative Multi-hop Transmission With and Without Presence of Hardware Impairments. Entropy.

[8] Tin P.T., Nam P.M., Duy T.T., Phuong T.T., Voznak M. (2019). Secrecy Performance of TAS/SC-based Multi-hop Harvest-to-Transmit Cognitive WSNs under Joint Constraint of Interference and Hardware Imperfection. Sensors.

[9] Zhang T., Cai Y., Huang Y., Duong T.Q., Yang W. (2016). Secure Transmission in Cognitive MIMO Relaying Networks With Outdated Channel State Information. IEEE Access.

[10] Huang Y., Wang J., Zhong C., Duong T.Q., Karagiannidis G.K. (2016). Secure Transmission in Cooperative Relaying Networks with Multiple Antennas. IEEE Trans. Wirel. Commun..

[11] Yang M., Guo D., Huang Y., Duong T.Q., Zhang B. (2016). Secure Multiuser Scheduling in Downlink Dual-hop Regenerative Relay Networks over Nakagami-m Fading Channels. IEEE Trans. Wirel. Commun..

[12] Zhao R., Lin H., He Y.-C., Chen D.-H., Huang Y., Yang L. (2018). Secrecy Performance of Transmit Antenna Selection for MIMO Relay Systems with Outdated CSI. IEEE Trans. Commun..

[13] Mo J., Tao M., Liu L. (2012). Relay Placement for Physical Layer Security: A Secure Connection Perspective. IEEE Commun. Lett..

[14] Lee J.-H., Sohn I., Kim Y.-H. (2016). Transmit Power Allocation for Physical Layer Security in Cooperative Multi-Hop Full-Duplex Relay Networks. Sensors.

[15] Keshav S., Ku M.-L., Biswas S., Ratnarajah T. (2017). Energy-Efficient Subcarrier Pairing and Power Allocation for DF Relay Networks with an Eavesdropper. Energies.

[16] Hieu T.D., Duy T.T., Kim B.-S. (2018). Performance Enhancement for Multi-hop Harvest-to-Transmit WSNs with Path-Selection Methods in Presence of Eavesdroppers and Hardware Noises. IEEE Sens. J..

[17] Cao K., Cai K., Wu Y., Yang W. (2017). Cooperative Jamming for Secure Communication with Finite Alphabet Inputs. IEEE Commun. Lett..

[18] Kang J.M., Yang J., Ha J., Kim I.M. (2017). Joint Design of Optimal Precoding and Cooperative Jamming for Multiuser Secure Broadcast Systems. IEEE Trans. Veh. Technol..

[19] Ma H., Cheng J., Wang X., Ma P. (2018). Robust MISO Beamforming with Cooperative Jamming for Secure Transmission From Perspectives of QoS and Secrecy Rate. IEEE Trans. Commun..

[20] Zhang G., Xu J., Wu Q., Cui M., Li X., Lin F. (2018). Wireless Powered Cooperative Jamming for Secure OFDM System. IEEE Trans. Veh. Technol..

[21] Nasir A.A., Zhou X., Durrani S., Kennedy R.A. (2013). Relaying Protocols for Wireless Energy Harvesting and Information Processing. IEEE Trans. Wirel. Commun..

[22] Atapattu S., Evans J. (2016). Optimal Energy Harvesting Protocols for Wireless Relay Networks. IEEE Trans. Wirel. Commun..

[23] Wang L., Wong K.K., Jin S., Zheng G., Heath R.W. (2018). A New Look at Physical Layer Security, Caching, and Wireless Energy Harvesting for Heterogeneous Ultra-Dense Networks. IEEE Commun. Mag..

[24] Chang S., Li J., Fu X., Zhang L. (2017). Energy Harvesting for Physical Layer Security in Cooperative Networks Based on Compressed Sensing. Entropy.

[25] Xu C., Zheng M., Liang W., Yu H., Liang Y.C. (2016). Outage Performance of Underlay Multihop Cognitive Relay Networks with Energy Harvesting. IEEE Commun. Lett..

[26] Xu C., Zheng M., Liang W., Yu H., Liang Y.C. (2017). End-to-end Throughput Maximization for Underlay Multi-hop Cognitive Radio Networks with RF Energy Harvesting. IEEE Trans. Wirel. Commun..

[27] Zhu G., Zhong C., Suraweera H.A., Karagiannidis G.K., Zhang Z., Tsiftsis T.A. (2015). Wireless Information and Power Transfer in Relay Systems with Multiple Antennas and Interference. IEEE Trans. Commun..

[28] Chen E., Xia M., Da Costa D., Aissa S. (2017). Multi-hop Cooperative Relaying with Energy Harvesting from Co-Channel Interferences. IEEE Commun. Lett..

[29] Liu M., Liu Y. (2017). Power Allocation for Secure SWIPT Systems with Wireless-Powered Cooperative Jamming. IEEE Commun. Lett..

[30] MacKay D. (2005). Fountain Codes. IEE Proc. Commun..

[31] Castura J., Mao Y. (2006). Rateless Coding over Fading Channels. IEEE Commun. Lett..

[32] Nguyen H.D.T., Tran L.N., Hong E.K. (2011). On Transmission Efficiency for Wireless Broadcast Using Network Coding and Fountain Codes. IEEE Commun. Lett..

[33] Yue J., Lin Z., Vucetic B. (2014). Distributed Fountain Codes With Adaptive Unequal Error Protection in Wireless Relay Networks. IEEE Trans. Wirel. Commun..

[34] Niu H., Iwai M., Sezaki K., Sun L., Du Q. (2014). Exploiting Fountain Codes for Secure Wireless Delivery. IEEE Commun. Lett..

[35] Li W., Du Q., Sun L., Ren P., Wang Y. Security Enhanced via Dynamic Fountain Code Design for Wireless Delivery. Proceedings of the IEEE 2016 IEEE Wireless Communications and Networking Conference.

[36] Sun L., Ren P., Du Q., Wang Y. (2016). Fountain-coding Aided Strategy for Secure Cooperative Transmission in Industrial Wireless Sensor Networks. IEEE Trans. Ind. Inform..

[37] Du Q., Xu Y., Li W., Song H. (2018). Security Enhancement for Multicast over Internet of Things by Dynamically Constructed Fountain Codes. Wirel. Commun. Mob. Comput..

[38] Hung D.T., Duy T.T., Trinh D.Q., Bao V.N.Q. Secrecy Performance Evaluation of TAS Protocol Exploiting Fountain Codes and Cooperative Jamming under Impact of Hardware Impairments. Proceedings of the 2nd International Conference on Recent Advances in Signal Processing, Telecommunications & Computing (SigTelCom).

[39] Hung D.T., Duy T.T., Trinh D.Q., Bao V.N.Q., Hanh T. Security-Reliability Analysis of Power Beacon-Assisted Multi-hop Relaying Networks Exploiting Fountain Codes with Hardware Imperfection. Proceedings of the International Conference on Advanced Technologies for Communications (ATC).

[40] Mokhtar M., Gomaa A., Al-Dhahir N. (2013). OFDM AF Relaying under I/Q Imbalance: Performance Analysis and Baseband Compensation. IEEE Trans. Commun..

[41] Björnson E., Matthaiou M., Debbah M. (2013). A New Look at Dual-Hop Relaying: Performance Limits with Hardware Impairments. IEEE Trans. Commun..

[42] Son P.N., Kong H.Y. (2017). Energy-Harvesting Decode-and-Forward Relaying under Hardware Impairments. Wirel. Pers. Commun..

[43] Solanki S., Upadhyay P.K., da Costa D.B., Bithas P.S., Kanatas A.G., Dias U.S. (2018). Joint Impact of RF Hardware Impairments and Channel Estimation Errors in Spectrum Sharing Multiple-Relay Networks. IEEE Trans. Commun..

[44] Zarei S., Gerstacker W.H., Aulin J., Schober R. (2017). Multi-Cell Massive MIMO Systems with Hardware Impairments: Uplink-Downlink Duality and Downlink Precoding. IEEE Trans. Wirel. Commun..

[45] Gradshteyn I.S., Ryzhik I.M. (2007). Table of Integrals, Series, and Products.

[46] Duy T.T., Alexandropoulos G.C., Vu T.T., Vo N.-S., Duong T.Q. (2016). Outage Performance of Cognitive Cooperative Networks with Relay Selection over Double-Rayleigh Fading Channels. IET Commun..

